# The Mucus-Binding Factor Mediates *Lacticaseibacillus rhamnosus* CRL1505 Adhesion but Not Immunomodulation in the Respiratory Tract

**DOI:** 10.3390/microorganisms12061209

**Published:** 2024-06-16

**Authors:** Binghui Zhou, Mariano Elean, Lorena Arce, Kohtaro Fukuyama, Kae Tomotsune, Stefania Dentice Maidana, Sudeb Saha, Fu Namai, Keita Nishiyama, María Guadalupe Vizoso-Pinto, Julio Villena, Haruki Kitazawa

**Affiliations:** 1 Laboratory of Animal Food Function, Graduate School of Agricultural Science, Tohoku University, Sendai 980-8572, Japan; binghui.zhou.e2@tohoku.ac.jp (B.Z.); kotaro.fukuyama.p8@dc.tohoku.ac.jp (K.F.); kaetomotsune@gmail.com (K.T.); fu.namai.a3@tohoku.ac.jp (F.N.); keita.nishiyama.a6@tohoku.ac.jp (K.N.); 2 Livestock Immunology Unit, International Education and Research Center for Food Agricultural Immunology, Graduate School of Agricultural Science, Tohoku University, Sendai 980-8572, Japan; 3 Laboratory of Immunobiotechnology, Reference Centre for Lactobacilli (CERELA-CONICET), Tucuman CP4000, Argentina; melean@cerela.org.ar (M.E.); stefi.dentice@gmail.com (S.D.M.); 4 Infection Biology Laboratory, INSIBIO (CONICET-UNT), Tucuman CP4000, Argentina; lore.arce.oaq@gmail.com (L.A.);; 5 Department of Dairy Science, Faculty of Veterinary, Animal and Biomedical Sciences, Sylhet Agricultural University, Sylhet 3100, Bangladesh

**Keywords:** nasal probiotics, mucus-binding factor mutant, *Lacticaseibacillus rhamnosus* CRL1505, respiratory immunity, porcine bronchial epithelial cell, antiviral immunity, alveolar macrophages

## Abstract

*Lacticaseibacillus rhamnosus* CRL1505 possesses immunomodulatory activities in the gastrointestinal and respiratory tracts when administered orally. Its adhesion to the intestinal mucosa does not condition its beneficial effects. The intranasal administration of *L. rhamnosus* CRL1505 is more effective than the oral route at modulating immunity in the respiratory tract. Nonetheless, it has not yet been established whether the adherence of the CRL1505 strain to the respiratory mucosa is needed to provide the immune benefits to the host. In this study, we evaluated the role of adhesion to the respiratory mucosa of the mucus-binding factor (mbf) knock-out *L. rhamnosus* CRL1505 mutant (*Δmbf* CRL1505) in the context of a Toll-like receptor 3 (TLR3)-triggered innate immunity response. In vitro adhesion studies in porcine bronchial epitheliocytes (PBE cells) indicated that *L. rhamnosus Δmbf* CRL1505 adhered weakly compared to the wild-type strain. However, in vivo studies in mice demonstrated that the *Δmbf* CRL1505 also reduced lung damage and modulated cytokine production in the respiratory tract after the activation of TLR3 to a similar extent as the wild-type strain. In addition, the mutant and the wild-type strains modulated the production of cytokines and antiviral factors by alveolar macrophages in the same way. These results suggest that the Mbf protein is partially involved in the ability of *L. rhamnosus* CRL1505 to adhere to the respiratory epithelium, but the protein is not necessary for the CRL1505 strain to exert its immunomodulatory beneficial effects. These findings are a step forward in the understanding of molecular interactions that mediate the beneficial effects of nasally administered probiotics.

## 1. Introduction

Probiotics are defined as “live microorganisms that confer a health benefit on the host when administered in adequate amounts” [1]. Several bacteria and yeast genera have been reported to confer beneficial properties, including members of the lactobacilli family, as well as *Bifidobacterium* spp., *Enterococcus* spp., *Streptococcus* spp., *Propionibacterium* spp., *Bacillus cereus*, *Escherichia coli*, and *Saccharomyces boulardii*. In humans and in certain mammals such as pigs, lactobacilli are part of the microbiota of the oral cavity, gastrointestinal tract, respiratory tract, vagina, and skin [2,3]. Certain strains, which are consumed as part of the diet in the form of functional foods, may persist for a limited period in the gastrointestinal tract. These beneficial strains may adhere to the epithelium and thereby stimulate health-promoting immune responses in host intestinal cells or competitively displace invading pathogens [4].

Immunomodulatory probiotics represent a natural alternative to help control bacterial and viral infections. Their mechanism is based on activating and modulating the immune system of the host in such a way that it can respond faster and more effectively to a pathogen without an exacerbated inflammation [2]. For instance, Gabryszewski et al. [5] showed a remarkable efficacy of intranasal lactobacilli priming in dampening inflammation triggered by viruses and providing robust protection against severe illness. In these series of experiments, priming the respiratory tract with *Lactiplantibacillus plantarum* fostered heterologous immunity and led to complete protection against the severe consequences of a subsequent acute respiratory virus infection. This protection was achieved through significant suppression of the inflammatory response against the virus and reduced viral presence in lung tissue [6,7]. Similarly, *Lacticaseibacillus rhamnosus* CRL1505 has long been shown to have diverse immunomodulatory effects on the respiratory mucosae in vivo, especially enhancing antiviral immunity while at the same time regulating inflammation [8,9,10]. Some of these studies have also shown that the viability of the CRL1505 strain administered nasally is not a necessary condition to exert its beneficial effects since heat-killed bacteria can also modulate the respiratory immunity [8,9,10]. These results would indicate that the adhesion and colonization of the respiratory mucosa would not be necessary for the immunomodulatory effects of *L. rhamnosus* CRL1505, but this hypothesis has not been investigated.

It was reported that *L. rhamnosus* Kx151A1, *Limosilactobacillus reuteri* PTA-5289, and *Ligilactobacillus salivarius* LMG9477 can interfere in the binding of *S. pyogenes* to epithelial cells of the pharynx [11]. Interestingly, there is also competition towards viral receptors: by binding to viral receptors, lactobacilli hinder virus entry to the host cell. Further, some cell wall components such as lipopeptides have been found to inhibit the binding of SARS-CoV-2 spike glycoprotein by competing with angiotensin-converting enzyme 2 [12]. In addition, it was shown that *Lacticaseibacillus casei* AMBR2 can successfully colonize the nasal epithelium of healthy volunteers and inhibit the growth of respiratory pathogens [13]. The AMBR2 strain contains fimbriae that enable strong adherence to the respiratory epithelium. These works suggests that lactobacilli have the ability to adhere to and colonize the respiratory tract.

The *L. rhamnosus* GG genome encodes for adhesins to mucins and intestinal epithelial cells, including the adhesin MabA, fimbriae, and the mucus-binding factor (Mbf) [14]. The Mbf structure contains an N-terminal 4 Pfam-MucBP (mucin-binding protein) domain and a C-terminal LPXTG motif that participate in the adhesion to the extracellular matrix. The main adhesins for mucins and intestinal epithelial cells in *L. rhamnosus* GG are fimbriae and MabA, while the Mbf proteins presumably have auxiliary roles. For *L. rhamnosus* LC705, it has been hypothesized that Mbf constitutes one of the key mucosal adhesins, but it was shown that this protein has a minor role in the total mucus-binding capacity in the GG strain [15]. On the other hand, Nishiyama et al. [15] showed that the adherence of *L. rhamnosus* GG to the extracellular matrix is mainly mediated by the Mbf protein. Thus, the role of the Mbf protein in adhesion to the intestinal mucosa depends on the strain of lactobacilli under study.

Recently, we showed that the *L. rhamnosus* CRL1505 *mbf* knock-out mutant (*Δmbf* CRL1505) preserved its immunomodulatory functions and was still able to adhere to both mucins and porcine intestinal epithelial (PIE) cells. However, the adhesive abilities of the *Δmbf* CRL1505 strain were lower than those of the wild type (WT) [16]. The role of Mbf-containing proteins has been studied in lactobacilli whose natural habitat is the gastrointestinal tract, but the involvement of these proteins in the adhesion of lactobacilli to the respiratory mucosa has not been studied. The aim of this work was to investigate the role of Mbf in the adhesion of *L. rhamnosus* CRL1505 to the respiratory tract and in its capacity to modulate the respiratory innate antiviral immune response. Then, the adhesion of *L. rhamnosus* CRL1505 and *Δmbf* CRL1505 strains to bronchial epithelial cells was comparatively evaluated in vitro while their immunomodulatory activities in the respiratory tract were assessed in vivo.

## 2. Materials and Methods

### 2.1. Microorganisms

*L. rhamnosus* CRL1505 and *Lactiplantibacillus plantarum* CRL1506 were obtained from the CERELA culture collection (Tucumán, Argentina). *L. rhamnosus Δmbf* CRL1505 is a mucus-binding protein knock-out that was constructed and phenotypically checked by Tomotsune et al. [16]. The *L. plantarum* MPL16 and *L. salivarius* FFIG58 strains were obtained from the Food and Feed Immunology Group–Tohoku University culture collection (Sendai, Japan). Lactobacilli were cultured overnight at 37 °C in Man–Rogosa–Sharpe broth (MRS, Oxoid, Hampshire, UK). Bacteria were kept freeze-dried at −70 °C. Before administration, the cells were harvested by centrifugation at 3000× *g* for 10 min, washed three times with sterile PBS (pH 7.2), and finally resuspended in the same buffer.

### 2.2. Adhesion Assays

The micro-plate method with fluorescent bacteria [17] was used to evaluate the adhesion of lactobacilli to porcine bronchial epithelial (PBE) cells. PBE cells were seeded on a collagen-coated 24-well plate (MS0024, Sumitomo Bakelite, Tokyo, Japan) in Dulbecco’s modified Eagle medium (DMEM, GIBCO, NY, USA), supplemented with 10% FBS, penicillin (10 U/mL), and streptomycin (10 μg/mL). Cell counts were determined by blood cell counting after 6 days. Then, PBE cells were transferred to a type I collagen-coated 96-well cell culture plate at a density of 5000 cells/well and cultured for 5 days for adhesion experiments. Bacteria were cultured and washed with PBS (6000 rpm, 10 min, three times), and the pellet was suspended in PBS containing 1 mM of carboxyfluorescein diacetate (CFDA). The bacterial cells were incubated at 37 °C for 1 h and then washed with PBS (6000 rpm, 10 min, three times) to remove CFDA on the microbial surface. A hemocytometer was used to count fluorescent bacteria. Fluorescent lactobacilli were added to PBE cells at 100 UFC per epithelial cell and the cocultures were incubated for 48 h. Non-adherent bacteria were washed out with PBS and PBE cells were lysed with 0.1 N NaOH at 60 °C. The fluorescence was measured using a 2030 ARVO Multilabel Reader (Perkin Elmer, Fukuoka, Japan).

### 2.3. Animals and Treatments

Adult (6-week-old) BALB/c mice were obtained from CERELA-CONICET (Tucumán, Argentina). The mice were housed in plastic cages in a room with a controlled temperature and 12 h light/darkness cycles. Each experimental group consisted of 5–6 mice fed a conventional balanced diet ad libitum. *L. rhamnosus* strains (10^8^ cells/mouse/day) were administered via nostrils to infant mice for three consecutive days before being challenged with poly(I:C). Lightly anesthetized mice were administered 250 μg poly(I:C) in 50 µL PBS (equivalent to 10 mg/kg body weight) via nostrils dropwise for three consecutive days. Control animals received the same volume of PBS. Experiments with animals were performed in accordance with the guide for the care and use of laboratory animals and were approved by the CERELA-CONICET Animal Care and Ethics Committee. Approval Code: BIOT-CRL/19 protocol. Approval Date: Dec 2019.

### 2.4. Lung Injury Parameters

Broncho-alveolar lavages (BALs) were taken as reported previously [10]. Briefly, two sequential lavages were taken by injecting sterile PBS through the intubated trachea. BAL samples were centrifuged for 10 min at 900× *g* and kept frozen at −70 °C until analysis. The albumin content was determined colorimetrically using a commercial kit (Wiener Lab, Buenos Aires, Argentina). Lactate dehydrogenase (LDH) activity was determined by measuring the formation of the reduced form of nicotinamide adenine dinucleotide (NAD) using a commercial kit and following the manufacturer’s instructions (Wiener Lab).

### 2.5. Flow Cytometry Analysis

Lung single cells were obtained as described previously [10]. Briefly, lungs were excised, minced, and incubated for 90 min in 15 mL of RPMI 1640 medium (Sigma, Tokyo, Japan) containing 300 U of collagenase (Yakult Honsha Co., Tokyo, Japan). The samples were gently tapped into a plastic dish in order to dissociate the lung tissue into single cells. Hypotonic lysis was used to deplete erythrocytes and the single cells were washed with RPMI medium supplemented with 10% heat-inactivated fetal calf serum. Cells were counted using Trypan Blue exclusion, adjusted to 5 × 10^6^ cells/mL, and preincubated with anti-mouse CD32/CD16 monoclonal antibody (Fc block) for 15 min at 4 °C. Then, the cells were incubated with different antibody mixes for 30 min at 4 °C and washed with FACS buffer. Staining was conducted with fluorochrome-conjugated antibodies against CD11c (APC), SiglecF (PE) (BD Bioscience, San Jose, CA, USA), CD45 (FITC) (eBioscience), and MHC-II (PerCP) (Thermo Fisher Scientific, Waltham, MA, USA). Cells were then acquired on a BD FACSCaliburTM flow cytometer (BD Biosciences), and data were analyzed with FlowJo software (TreeStar).

### 2.6. Alveolar Macrophage (AM) Primary Cultures

Primary cultures of murine AMs were performed as described by Clua et al. [9]. Macrophages were obtained from infant mice via BAL samples by flushing with 1 mL of warm, sterile PBS to which 5 mM EDTA was added. After two washing steps, the AMs were resuspended in RPMI 1640 medium, with 10% FBS, 1 mM L-glutamine, and 100-U/mL penicillin-streptomycin. AMs were seeded in 24-well plates (10^5^ cells/well) and incubated at 37 °C and 5% CO_2_ for 2 h to allow adherence. Non-adherent cells were washed out, and adherent AMs were maintained in culture in RPMI 1640 medium with 10% FBS, 1-mM L-glutamine, and 100-U/mL penicillin-streptomycin at 37 °C and 5% CO_2_ for 24 h before stimulation. AMs were stimulated with *L. rhamnosus* CRL1505 WT or the Δ*mbf* CRL1505 mutant (50 UFC per epithelial cell) for 24 h, and control wells received PBS without bacteria. The supernatants were collected for cytokine analysis after 24 h.

### 2.7. Cytokine Quantification in BAL and Cell Culture Supernatants

All cytokines were measured in BAL and AM culture supernatants using commercially available ELISA kits following the manufacturer´s instructions (R&D Systems, MN, USA). The analyzed cytokines are the following: IFN-β (Mouse IFN-beta enzyme-linked immunosorbent assay (ELISA) Kit), IFN-γ (Mouse IFN-gamma Quantikine ELISA Kit), IL-6 (Mouse IL-6 Quantikine ELISA Kit), IL-10 (Mouse IL-10 Quantikine ELISA Kit), IL-12 (Mouse IL-12 p70 DuoSet ELISA), and IL-27 (Mouse IL-27 p28/IL-30 Quantikine ELISA Kit).

### 2.8. Determination of Antiviral Factors and Cytokines Expression

Two-step real-time quantitative PCR (qPCR) was used to evaluate changes in the expression of the antiviral factors IFN-α, IFN-β, IFN-γ, Mx1, RNAseL, and OAS1, as well as the cytokines TNF-α, IL-1α, IL-1β, IL-6, IL-10, and IL-27 in AMs after 12 h of poly(I:C) stimulation. TRIzol reagent (Invitrogen) was used for total RNA isolation and a Quantitect Reverse-Transcription (RT) Kit (Qiagen, Tokyo, Japan) was used for the synthesis of cDNAs, following the instructions of the manufacturers. The qPCR was carried out with a 7300 Real-Time PCR System (Applied Biosystems, Warrington, UK) and the Platinum SYBR Green qPCR SuperMix uracil-DNA glycosylase (UDG) with 6-carboxyl-X-rhodamine (ROX) (Invitrogen, Carlsbad, CA, USA). The primers for antiviral factors and cytokines were described previously [9]. The following steps were used in PCR cycling: 2 min at 50 °C, 2 min at 95 °C, 40 cycles of 15 s at 95 °C, 30 s at 60 °C, and 30 s at 72 °C. The expression of β-actin was used to normalize the cDNA levels as described before [9].

### 2.9. Statistical Analysis

All experiments were conducted in triplicate and the results are expressed as the mean ± standard deviation (SD). Two-way ANOVA followed by Tukey’s test (for pairwise comparisons of the means) were used to test for differences between the groups (*p* < 0.05).

## 3. Results

### 3.1. Adhesion of L. rhamnosus CRL1505 and the Δmbf CRL1505 Mutant to Porcine Bronchial Epithelial Cells

**Figure 1:**
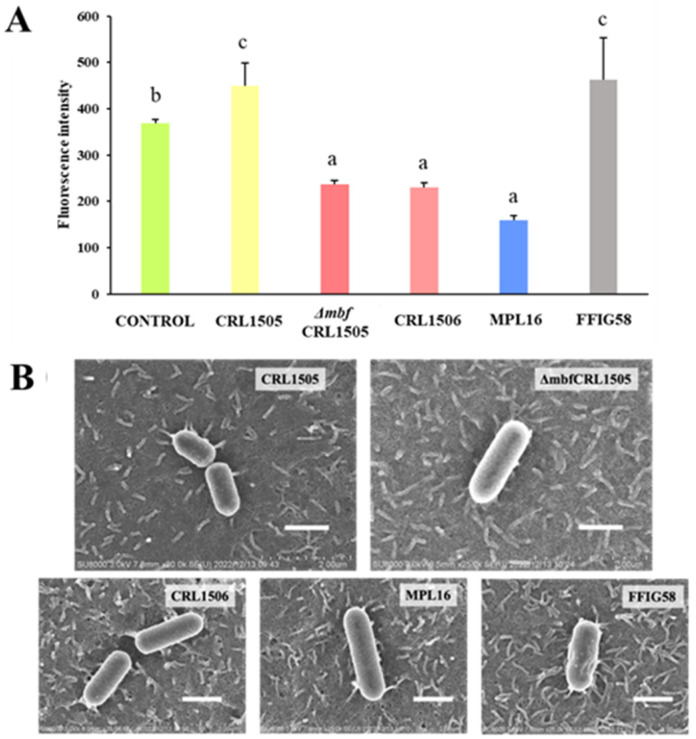
(**A**) Adhesion of wild-type *Lacticaseibacillus rhamnosus* CRL1505 and *L. rhamnosus Δmbf* CRL1505 to PBE cells. Data from three independent experiments are shown. Different letters indicate statistically significant differences (*p* ≤ 0.05). (**B**) Scanning electron microscope (SEM) analysis of wild-type *L. rhamnosus* CRL1505, *L. rhamnosus Δmbf* CRL1505, *Lactiplantibacillus plantarum* CRL1506, *L. plantarum* MPL16, and *Ligilactobacillus salivarius* FFIG58 adhering to PBE cells. Scale bar: 1 μm.

We evaluated if lactobacilli adhere to porcine bronchial epithelial cells. Porcine-derived cells are a good substitute for human cells because they are not transformed cells and do not present malign features; they mimic both the physical and functional characteristics of the respiratory tract [18,19]. The five strains evaluated here presented different adhesion profiles. The most adherent strains were *L. rhamnosus* CRL1505 and *L. salivarius* FFIG58, whereas *L. plantarum* CRL1506 and MPL16 were less adherent (Figure 1A).

We also assayed the adhesion capacity of the *Δmbf* CRL1505 mutant and saw that although the strain was able to adhere, the adhesion values decayed significantly compared to those of the WT strain (*p* < 0.05) (Figure 1A). Thus, although Mbf plays an important role in *L. rhamnosus* CRL1505 adhesion to epithelial cells, there are more factors involved in this process. Further, scanning electron microscope (SEM) micrographs show that *L. rhamnosus* CRL1505 has a rough surface with appendix-like structures that interact with the cilia present on the surface of the epithelial cells (Figure 1B). In contrast, the *Δmbf* CRL1505 mutant has a smooth surface, and this interaction is no longer evident (Figure 1B). *L. salivarius* FFIG58, which has similar adhesion values to those of *L. rhamnosus* CRL1505, also presents a rough surface, while the less adherent strains *L. plantarum* CRL1506 and MPL16 have a smooth surface (Figure 1B).

### 3.2. L. rhamnosus CRL1505 and the Δmbf CRL1505 Mutant Reduce the Lung Damage Produced by poly(I:C) In Vivo

**Figure 2:**
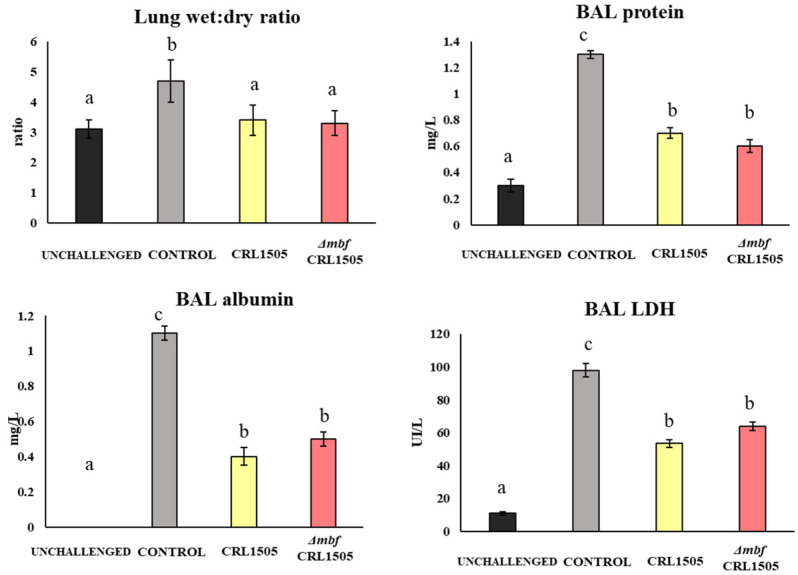
Immunomodulation of wild-type *Lacticaseibacillus rhamnosus* CRL1505 and *L. rhamnosus Δmbf* CRL1505 stains on the respiratory innate antiviral immune response triggered by TLR3 activation. Balb/c mice (6-week-old) were nasally treated with the wild-type *L. rhamnosus* CRL1505 or *L. rhamnosus Δmbf* CRL1505 (10^8^ cells/mouse) for three consecutive days prior to the nasal stimulation with poly(I:C). Control mice received the same volume of PBS. Lung wet:dry weight and BAL proteins, LDH, and albumin were determined 2 days after the poly(I:C) stimulation. Data from three independent experiments are shown. Different letters indicate statistically significant differences (*p* < 0.05).

During three consecutive days, mice received *L. rhamnosus* CRL1505 or the *Δmbf* CRL1505 mutant via nostrils. On the fourth day, animals were stimulated with poly(I:C), a TLR3 analogue. As expected, lungs suffered from inflammation as reflected by a high lung wet:dry ratio, an elevated protein content (especially albumin) in BAL, and an elevated concentration of LDH (a biomarker of cell damage) (Figure 2).

All parameters were significantly reduced if mice received the lactobacilli treatment before challenge (*p* < 0.05) (Figure 2). There were no significant differences in the way in which the *Δmbf* CRL1505 mutant modulated these parameters compared to the WT strain, indicating that the immunomodulatory effect is independent of the Mbf protein.

### 3.3. L. rhamnosus CRL1505 and the Δmbf CRL1505 Mutant Modulate Cytokine Production in BAL

**Figure 3:**
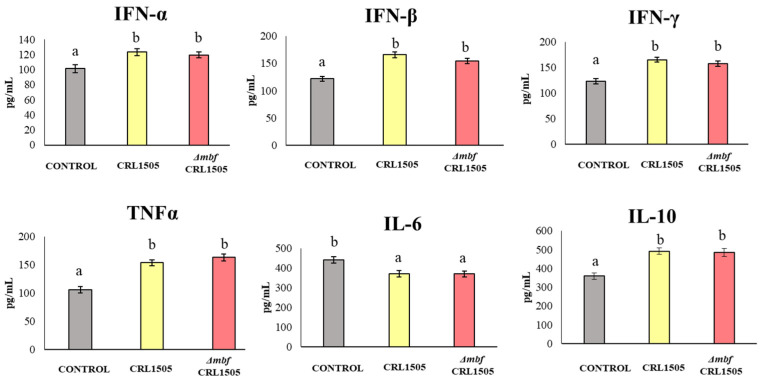
Immunomodulatory capacities of wild-type *Lacticaseibacillus rhamnosus* CRL1505 and *L. rhamnosus* Δ*mbf* CRL1505 strain on the respiratory innate antiviral immune response triggered by TLR3 activation. Balb/c mice (6-week-old) were nasally treated with the wild-type *L. rhamnosus* CRL1505 *or L. rhamnosus Δmbf* CRL1505 (10^8^ cells/mouse) for three consecutive days prior to the nasal administration of poly(I:C). Untreated mice challenged with poly(I:C) were used as controls. The numbers of neutrophils, TNF-α, IL-6, and IL-8 in BAL samples were determined 2 days after the poly(I:C) stimulation. Data from three independent experiments are shown. Different letters indicate statistically significant differences (*p* < 0.05).

Control mice treated with poly(I:C) that did not receive lactobacilli before the challenge had high levels of IL-6 in their lungs, which were reduced if mice were previously treated with either *L. rhamnosus* CRL1505 or the *Δmbf* CRL1505 mutant (*p* < 0.05) (Figure 3). In contrast, interferons (IFN-γ, IFN-β, IFN-α), TNF-α, and the regulatory cytokine IL-10 significantly increased if mice were pretreated with both lactobacilli. There were no significant differences between the WT strain and the *Δmbf* CRL1505 mutant.

### 3.4. L. rhamnosus CRL1505 and the Δmbf CRL1505 Mutant Modulate Lung Immune Cells after poly(I:C) Challenge

**Figure 4:**
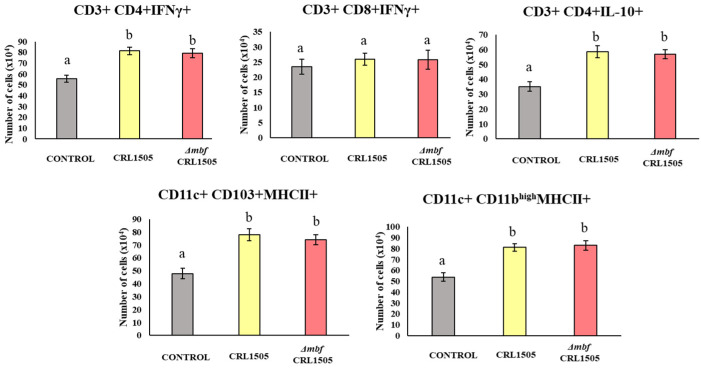
Immunomodulatory capacities of wild-type *Lacticaseibacillus rhamnosus* CRL1505 and *L. rhamnosus Δmbf* CRL1505 strain on the respiratory innate antiviral immune response triggered by TLR3 activation. Balb/c mice (6-week-old) were nasally treated with the wild-type *L. rhamnosus* CRL1505 or *L. rhamnosus Δmbf* CRL1505 (10^8^ cells/mouse) for three consecutive days prior to the nasal administration of TLR3 agonist poly(I:C). Untreated mice challenged with poly(I:C) were used as controls. The numbers of lung CD3^+^CD4^+^IFNγ^+^, CD3^+^CD4^+^IL-10^+^, CD11c^+^CD11b^high^MHC-II^+^, and CD11c^+^CD103^+^MHC-II^+^ were determined 2 days after the poly(I:C) stimulation. Data from three independent experiments are shown. Different letters indicate statistically significant differences (*p* < 0.05).

Both *L. rhamnosus* CRL1505 and the *Δmbf* CRL1505 mutant modified the number of immune cells in lungs. The CD3^+^CD4^+^IFNγ^+^, CD3^+^CD4+IL-10^+^, CD11c^+^CD11b^high^MHC-II^+^, and CD11c+CD103^+^MHC-II^+^ immune cell populations were detected in lungs in significantly higher numbers when mice received the lactobacilli before challenge with poly(I:C) (*p* < 0.05), whereas the CD3^+^CD8^+^IFNγ^+^ population remained unchanged compared to that of the untreated control (Figure 4). The effect of the *Δmbf* CRL1505 mutant was not different from that of the WT, suggesting that the Mbf protein does not participate in the modulation of these immune cell populations.

### 3.5. Effect of L. rhamnosus CRL1505 and the Δmbf CRL1505 Mutant on the AMs Cytokine and Antiviral Factors Response to poly(I:C)

**Figure 5:**
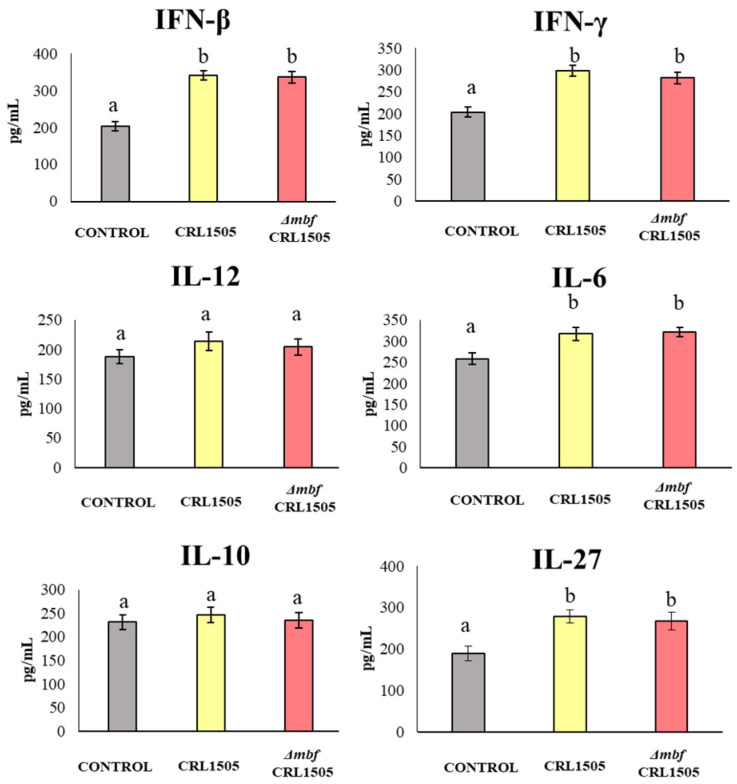
Immunomodulatory capacities of wild-type *Lacticaseibacillus rhamnosus* CRL1505 and *L. rhamnosus Δmbf* CRL1505 strain on the respiratory innate antiviral immune response triggered by TLR3 activation. Alveolar macrophages isolated from the wild type and the *Δmbf* CRL1505 mutant were challenged in vitro with the TLR3 agonist poly(I:C). IFN-β, IFN-γ, Il-12, IL-6, IL-10, and IL-27 levels were analyzed by ELISA after 24 h of TLR3 activation. Non-lactobacilli-treated alveolar macrophages challenged with poly(I:C) were used as controls. Data from three independent experiments are shown. Different letters indicate statistically significant differences (*p* < 0.05).

The AMs were isolated from the mice nasally treated with *L. rhamnosus* CRL1505 and the *Δmbf* CRL1505, as well as from the untreated controls, and they were cultivated and stimulated with poly(I:C) in vitro. The cytokines produced in response to this stimulus were quantified by ELISA in the supernatants. Figure 5 shows that IFN-β and IFN-γ, as well as IL-6 and IL-27, were produced at higher levels in AMs derived from animals treated with lactobacilli (*p* < 0.05). Both the WT CRL1505 strain and the *Δmbf* CRL1505 mutant produced the same effect. The other cytokines tested, namely IL-12 and IL-10, did not differ significantly from the untreated control (Figure 5).

**Figure 6:**
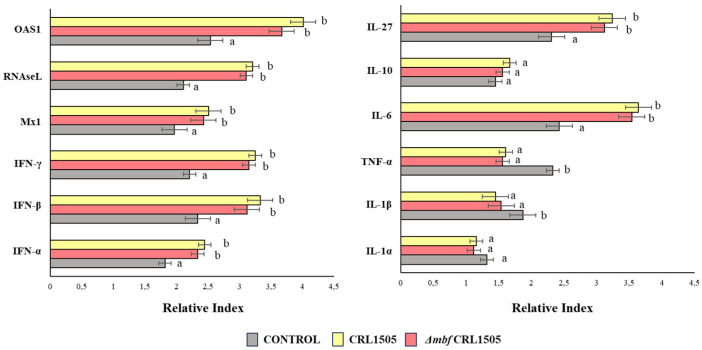
Immunomodulatory capacities of wild-type *Lacticaseibacillus rhamnosus* CRL1505 and *L. rhamnosus Δmbf* CRL1505 strain on the respiratory innate antiviral immune response triggered by TLR3 activation. Alveolar macrophages isolated from the wild type and the *Δmbf* CRL1505 mutant were challenged in vitro with the TLR3 agonist poly(I:C). The expression of cytokines and antiviral factors were analyzed by qPCR after 12 h of TLR3 activation. Non-lactobacilli-treated alveolar macrophages challenged with poly(I:C) were used as controls. Data from three independent experiments are shown. Different letters indicate statistically significant differences (*p* < 0.05).

The expressions of cytokines and antiviral factors in AMs isolated from the mice nasally treated with *L. rhamnosus* CRL1505 and *Δmbf* CRL1505, cultivated and stimulated with poly(I:C) in vitro, were also evaluated (Figure 6).

The expressions of the *IFN-α*, *IFN-β*, *IFN-γ*, *Mx1*, *RNAseL*, and *OAS1* antiviral factors in AMs isolated from mice nasally primed with *L. rhamnosus* CRL1505 and the *Δmbf* CRL1505 mutant were higher than those of the controls (*p* < 0.05), while no significant differences were observed between the lactobacilli treatments (Figure 6). Similarly, when the expressions of *TNF-α*, *IL-1β*, *IL-6*, and *IL-27* were analyzed, it was observed that all these cytokines were higher in *L. rhamnosus* CRL1505 and the *Δmbf* CRL1505 mutant groups compared to the controls (*p* < 0.05). The other cytokines tested, namely *IL-1α* and *IL-10*, did not differ significantly from the untreated control (Figure 6).

## 4. Discussion

Probiotic bacteria adhere to the extracellular matrix and to intestinal epithelial cells, a characteristic that has been related to their ability to colonize or at least persist for longer periods of time in the gut. Furthermore, adhesion is considered an important characteristic of probiotic strains, and therefore this property is usually evaluated in the screening of potential beneficial microorganisms for the intestinal tract. However, the importance of adhesion has not been studied for probiotics applied nasally. Previously, we showed that *L. rhamnosus* CRL1505 administered nasally is able to modulate the respiratory innate immune system allowing an enhanced resistance to viral and bacterial infections [8,9]. We speculated that the adhesion and colonization of the respiratory mucosa would not be necessary for the CRL1505 strain to exert its immunomodulatory effects since heat-killed bacteria and purified peptidoglycan preserved the capacity to modulate the innate immune response in a way similar to that of live bacteria [8,9]. In this work, we tested this hypothesis, evaluating whether *L. rhamnosus* CRL1505 was able to adhere to respiratory epithelial cells and if this ability was a condition for exerting its immunomodulation. Our studies focused on Mbf, considering that this protein is involved in the adhesion of probiotic lactobacilli to mucus and components of the extracellular matrix [15], and that the major constituents of the mucus coating the respiratory epithelium are mucins, a family of high-molecular-weight glycosylated proteins [20].

The results presented here show that Mbf of *L. rhamnosus* CRL1505 (a) is involved in the ability of the strain to adhere to bronchial epithelial cells and (b) has no role in its immunomodulatory capacity in the respiratory tract.

(a) *Mbf of L. rhamnosus CRL1505 participates in the adhesion to bronchial epithelial cells*. The Mbf protein is one of the key mucosal adhesins for *L. rhamnosus* LC705, but for *L. rhamnosus* GG it is only of importance for the adhesion to the extracellular matrix [15]. It was shown that the *mbf*-deficient *L. rhamnosus* FSMM22 considerably lost its ability to adhere to the extracellular matrix in vitro compared to the WT strain [15]. CRL1050 Mbf is highly homologous to Mbf of *L. rhamnosus* GG [21]. Recently, we showed that the Mbf protein of *L. rhamnosus* CRL1505 contributed to adhesion to the extracellular matrix and to porcine intestinal cells [16]. These previous works clearly demonstrated the role of the Mbf protein in adhesion of lactobacilli to the intestinal mucosa, although its involvement in the adhesion to the respiratory tract was not investigated before. To the best of our knowledge, the present study is the first demonstration of the role of Mbf in the adhesion of a probiotic bacteria to respiratory epithelial cells. We found that adhesion to bronchial epithelial cells in vitro was strongly mediated by Mbf, although it was not the only factor responsible. We observed that although the adhesion values of the *Δmbf* CRL1505 mutant decayed significantly compared to those of the WT strain, it also adhered to bronchial epithelial cells. It was found that other structures and molecules, such as pili appendages, glycolytic enzymes, and surface layer proteins like the MapA protein, can mediate the adhesion of probiotic lactobacilli [4]. Investigating the role of such molecules in the adhesion of *L. rhamnosus* CRL1505 to the respiratory epithelium is an interesting topic for future research.

(b) *Mbf of L. rhamnosus CRL1505 does not participate in its immunomodulatory activities in the respiratory tract.* Our research group thoroughly studied the immunomodulatory ability of nasally administered *L. rhamnosus* CRL1505. This strain beneficially regulates the TLR3-mediated respiratory innate immune response and reduces the local inflammatory tissue damage [9,10]. Our previous results and the ones presented here showed that AMs are a key immune cell population for the beneficial effects induced by the CRL1505 strain. AMs are the first immune cells that encounter pathogens in the alveolar spaces; as such, they initiate and regulate effector responses against them [22]. AMs can produce type I IFNs (IFN-β) and IFN-γ, which act on themselves and on neighboring cells, increasing the expression of hundreds of antiviral genes that improve the response of infected cells to a viral attack [23]. IFNs also help to coordinate the cellular antiviral response by activating recruited CD11c^+^MHC-II^+^ antigen-presenting cells and T cells that eliminate virus-infected cells [23]. Nasally administered *L. rhamnosus* CRL1505 enhances the production of IFN-β and IFN-γ, the expression of antiviral factors in AMs, and the numbers of lung CD11c^+^MHC-II^+^ cells and CD3^+^CD4^+^IFNγ^+^ T cells in response to TLR3 activation. In line with these immunological changes in the respiratory tract, it was shown that the CRL1505 strain improves the resistance of mice to influenza virus and syncytial respiratory virus [8,9], and reduces the severity of respiratory viral infections in children [10]. On the other hand, AMs actively participate in the regulation of inflammation by secreting cytokines like IL-27 and IL-6. It was demonstrated that IL-27 in combination with IL-6 regulates the induction of Treg IL-10^+^ cell maturation in the respiratory tract and, therefore, these cytokines are of importance in the protection against inflammatory damage during respiratory viral infections [24]. This mechanism of protection against lung damage in the context of TLR3 activation or respiratory virus infections is improved by *L. rhamnosus* CRL1505 since higher levels of lung CD3^+^CD4^+^IL-10^+^ T cells, IL-10, IL-27, and IL-6 are found in mice treated with lactobacilli when compared to controls [8,9].

Of note, in the experiments carried out in this study, we could not observe differences regarding modulation of the innate immune response triggered by poly(I:C) when *L. rhamnosus* CRL1505 and the *Δmbf* CRL1505 mutant were compared. Thus, the levels of BAL cytokines, as well as the lung immune cell populations, were similar independent of the strain used, i.e., WT or mutant. Furthermore, the *Δmbf* CRL1505 mutant strain was as effective as the WT *L. rhamnosus* CRL1505 at modulating the production of cytokines and the expressions of antiviral factors by AMs. Thus, the Mbf protein has no influence on the ability of *L. rhamnosus* CRL1505 to modulate TLR3-mediated respiratory immunity.

## 5. Conclusions

The in vitro and in vivo studies performed in this work with *L. rhamnosus* CRL1505 and the *Δmbf* CRL1505 mutant demonstrated that the Mbf protein is partially involved in the capacity of the probiotic lactobacilli to adhere to the respiratory epithelium, but it is not necessary for the CRL1505 strain to exert its immunomodulatory beneficial effects. These results are a step forward in the understanding of the molecular mechanisms involved in the beneficial effects of nasally administered probiotics.

## Data Availability

The original contributions presented in the study are included in the article; further inquiries can be directed to the corresponding author/s.
